# Biofilms in Infections of the Eye

**DOI:** 10.3390/pathogens4010111

**Published:** 2015-03-23

**Authors:** Paulo J. M. Bispo, Wolfgang Haas, Michael S. Gilmore

**Affiliations:** Departments of Ophthalmology, Microbiology and Immunology, Massachusetts Eye and Ear Infirmary, Harvard Medical School, Boston, MA, 02114 USA

**Keywords:** biofilm, eye, ocular infections, postoperative ocular infections, device-related ocular infections

## Abstract

The ability to form biofilms in a variety of environments is a common trait of bacteria, and may represent one of the earliest defenses against predation. Biofilms are multicellular communities usually held together by a polymeric matrix, ranging from capsular material to cell lysate. In a structure that imposes diffusion limits, environmental microgradients arise to which individual bacteria adapt their physiologies, resulting in the gamut of physiological diversity. Additionally, the proximity of cells within the biofilm creates the opportunity for coordinated behaviors through cell–cell communication using diffusible signals, the most well documented being quorum sensing. Biofilms form on abiotic or biotic surfaces, and because of that are associated with a large proportion of human infections. Biofilm formation imposes a limitation on the uses and design of ocular devices, such as intraocular lenses, posterior contact lenses, scleral buckles, conjunctival plugs, lacrimal intubation devices and orbital implants. In the absence of abiotic materials, biofilms have been observed on the capsule, and in the corneal stroma. As the evidence for the involvement of microbial biofilms in many ocular infections has become compelling, developing new strategies to prevent their formation or to eradicate them at the site of infection, has become a priority.

## 1. Introduction

Ever since Robert Koch and Louis Pasteur in the 1860’s established the modern field of bacteriology, studies employing pure bacterial cultures, often grown in liquid media (planktonic growth), have shaped our understanding of bacterial physiology and behavior. Pure cultures were required to establish microbial causes of disease, and growth in liquid media ensured that all cells were exposed to similar conditions and behaved in the same manner. As a result, most of the measures to control pathogenic bacteria (e.g., vaccines and antimicrobial agents) have been developed based on knowledge of bacteria grown as planktonic cells.

An appreciation for the fact that in nature, bacteria adhere to many abiotic or biotic surfaces and form communities of differentiated, interacting communities known as “biofilms”, emerged over the past few decades [1], and this concept was enthusiastically promoted by William (Bill) Costerton among others. Evidence of biofilm formation has been found in the analysis of microbial fossils including those from deep-sea hydrothermal sediments. This suggests that the ability to form biofilm is an ancient adaptation that dates back more than 3 billion years [2,3]. Biofilm formation conferred to individual bacteria the ability to collaborate and to adapt to a range of harsh environmental conditions, perhaps most of all, to evade predation by phagocytic microbes. The formation of a biofilm provides a microbe with a small measure of control over the local environment, including fluctuations in temperatures, pH, ultraviolet light, starvation, and exposure to toxic agents [4,5].

The ubiquity of biofilm formation in natural ecosystems, industrial systems, and medical settings has accelerated the pace of biofilm research. Advances in medical biofilm research have led to an understanding that biofilms represent the prevalent form of bacterial life during tissue colonization, and may occur in over 80% of microbial infections in the body [6]. Biofilms play important roles in human infections including native valve endocarditis, otitis media, chronic bacterial prostatitis, lung infections in patients with cystic fibrosis and periodontitis [7,8]. In addition, biofilms form on indwelling devices including prosthetic heart valves, coronary stents, intravascular catheters, urinary catheters, intrauterine devices, ventricular assist devices, neurosurgical ventricular shunts, prosthetic joint, cochlear implants, intraocular and contact lenses [7,8]. Due to their medical importance, development of anti-biofilm compounds for clinical use are of vital interest [9].

## 2. Microbial Biofilms

The very first description of a biofilm dates back to the 17th century when Anthony van Leeuwenhoek examined his own teeth scrapings with one of the first microscopes and found a large amount of small living “animalcules” in his dental plaque matter. He concluded in his report to the Royal Society of London in 1684 that the thick white material found between his teeth protected the bacteria embedded in this substance against the action of the vinegar that he used to wash his mouth [10]. At the time, miasmatic and humoral theories of disease were dominant, and it took an additional 200 years until the germ theory of disease was advanced by Robert Koch before a connection between microbes and disease was made.

Today, biofilms are generally defined as a community of sessile microbes held together by a polymeric extracellular matrix, adherent to a surface, interface or to other cells that are phenotypically distinct from their planktonic counterparts [8]. This definition, although reflective of many biofilms, is in our view restrictive, as there is no particular requirement that microbes be held together by an extracellular matrix as opposed to any other adherence principle (surface charge, a network of surface attached proteins, *etc.*), or that they even adhere to a surface (as a raft consisting of only microbes could achieve all of the behaviors usually ascribed to a biofilm, e.g., microbes transiting the lumen of the colon).

Members of a biofilm community, which can be of the same or multiple species, show varying stages of differentiation and exchange information, metabolites, and genes with each other. As a result, members of the biofilm community are in a diversity of physiologies influenced by the unequal sharing of nutrients and metabolic byproducts, which results in subpopulations with increased tolerance to antimicrobials and environmental stresses, the host immune system, and predatory microorganisms [8,11,12,13,14].

Canonically, biofilm development has been grouped into five stages that are reflective of conditions in many, but not all biofilms: (1) reversible aggregation of planktonic cells on a surface; (2) irreversible adhesion; (3) formation of microcolonies; (4) biofilm maturation; and (5) detachment and dispersion of cells [11,15]. The events that are of special significance for ocular infections and the treatment of biofilm infections will be discussed in greater detail below, while the reader is referred to several excellent reviews for details on other biofilm-related subjects [8,11,12,13,14,15,16].

In the established view of biofilm formation, planktonic cells initially adhere to a surface in a reversible, non-specific manner due to electrostatic interactions between the bacterial cell and the surface. Water contact angle measurements on bacterial cell lawns have shown that the surface of *P. aeruginosa* is highly hydrophobic, while *S. aureus* is highly hydrophilic [17]. Therefore, surface properties of a solid object can favor colonization by one microorganism over another.

Surfaces exposed to liquid solutions are generally coated with a conditioning layer consisting of molecules present in the solution. Antimicrobial agents that are present in multipurpose solutions, for example, bind non-specifically to the contact lenses [18]. Once the lens is placed on the surface of the eye, the disinfectant diffuses off the lens and is replaced by lipids and proteins present in the tear fluid [17,19,20]. The lens material plays an important role in this interaction, as it has been shown that hydrophilic contact lenses preferentially adsorb lysozyme from the tear film, while hydrophobic contact lenses accumulate more lipocalin and lactoferrin [17].

In addition to determining the local antimicrobial properties, this unique conditioning layer also provides specific anchor points for bacterial adhesion. Microbial adhesion to surfaces coated by proteins and other biomolecules is often accomplished by a class of molecules termed Microbial Surface Components Recognizing Adhesive Matrix Molecules (MSCRAMM), as well as other adhesive surface proteins [21]. As an example of the latter, in *S. epidermidis* and other staphylococci the bifunctional autolysin/adhesion protein AtlE, an abundant surface protein, mediates first attachment to abiotic surfaces and also matrix protein-covered devices [22].

In a moving suspension, cells are exposed to fairly uniform conditions. However, following attachment, the individual experience of a cell begins to differ from its neighbors (*i.e.*, a cell in the middle of a group will experience more excreted products and fewer factors from the environment than a cell on the periphery of a population), and as a result, cells begin to differentiate [23]. Many biofilms involve production of an extracellular matrix (ECM) that encases the cells, and in some cases, binds the cells together and that can be composed of polysaccharides, lipopolysaccharides, proteins, or extracellular DNA [24]. This process may be active or passive, in that cells on the surface of an adherent colony that are lysed by the ejection of neutrophil antimicrobial factors may encase and protect siblings below in a matrix consisting simply of cell lysate. Whatever the nature of the matrix, its chemical and physical properties contribute to the differentiation of cells within the encased population, a process that can protect the bacteria from the action of antimicrobial agents, host immune responses, bacteriophages and phagocytic amoeba [8]. In staphylococci, it appears that polysaccharide intercellular adhesin (PIA, encoded by the *icaADBC* locus), matrix proteins including the accumulation-associated protein (Aap) [25], and possibly the biofilm-associated homologue protein (Bhp, termed Bap in *S. aureus*) contribute to this matrix [26]. Commensal isolates of *S. epidermidis* and other coagulase-negative staphylococci (CoNS) recovered from healthy conjunctiva carry most of the genes related to biofilm maturation, suggesting that the ability to form biofilms is an integral part of their life-style [27,28,29].

As the microcolony grows through cell division or recruitment of more planktonic cells, the biofilm grows and takes on a three-dimensional structure that often includes open water channels [8,23]. Growing biofilms on various types of contact lenses have shown differences in cell densities and three-dimensional structures *in vitro*, suggesting an effect of the substrate on the development of the biofilm [30]. However, while several studies have measured biofilm thickness on various contact lens materials, with the underlying assumption that thicker biofilms are more likely to result in disease, the biological significance of these results remains unclear. In one experiment, Tam *et al.* [31] grew *P. aeruginosa* biofilms on custom contact lenses and tested them in a rat model of contact lens-associated keratitis [31]. Biofilms grown *in vitro* to low and to high cell densities both caused disease symptoms within 7–8 days, indicating that initial biofilm thickness did not matter [31]. In contrast, contaminated contact lenses that were transferred from one rat to a different healthy animal resulted in keratitis symptoms within 2 days [31]. These results suggest that adaptation to the host environment is a critical step in the pathogenesis of biofilm-related infections.

The three-dimensional organization of the biofilm causes gradients of oxygen, pH, and nutrients, resulting in the development of different microniches [32,33]. The cell’s individual physiological adaptations to these microniches results in physiological heterogeneity [13]. Cells near the surface of the biofilm will be exposed to more nutrients and oxygen and are therefore more metabolically active, while cells in the deep regions will be less active or even dormant. This heterogeneity results in a range of responses to antimicrobial agents, with metabolically active cells at the surface being rapidly killed while more internal, dormant cells are comparatively unaffected [32]. This, together with potential effects on diffusion of antimicrobial molecules within the biofilm, causes some cells in a biofilm to be recalcitrant to antimicrobial treatment, with antibiotic susceptibilities reduced by 10 to 1000-fold compared to planktonic counterparts [32].

The high local concentration of cells in a biofilm creates an ideal environment for information exchange through cell-to-cell communication and lateral gene transfer. Cell signaling mediated by secreted, accumulating messenger molecules, known as quorum sensing, allows bacteria to sense and respond to their environment and couple cell-density and other environmental cues with gene expression in ways that allow adaptive phenotypic responses. Quorum sensing has been shown to be involved in the control of biofilm formation and production of virulence and colonization factors in a variety of organisms of medical importance [34]. Cell-to-cell signaling is also involved in biofilm dispersion, which is of general and medical interest [35].

Bacterial cells can leave or be shed from the biofilm and revert to a planktonic life-style, often by degrading the ECM that holds the cells together [35]. For example, thermonuclease is a bacterial enzyme that degrades the extracellular DNA that holds *S. aureus* biofilms together, while alginate lyase degrades the alginate matrix important for *P. aeruginosa* biofilms [36,37]. These processes are coordinated by small signaling molecules that induce the expression of genes for biofilm dispersal. For example, *N*-butanoyl-l-homoserine lactone (C4HSL) belongs to the family of cell-density dependent autoinducers and has been implicated in the dispersal of *P. aeruginosa* biofilms [38]. Other small molecules implicated in biofilm dispersal include the *Pseudomonas* quinolone signal PQS, the furanosylborate autoinducer AI-2 from *Vibrio cholerae*, and the staphylococcal peptides δ-toxin and AIP-I [35].

## 3. Ocular Infections

The two leading causes of vision impairment worldwide are uncorrected refractive errors and cataract [39]. Measures to manage those eye abnormalities frequently include the use of contact lenses and the placement of intraocular lenses, and have enhanced the quality of life of millions of patients. Although use of such devices is of the utmost importance for correction of a variety of visual aberrations, they also provide a new surface on which many microbial pathogens can form biofilms (Table 1). As a result, device-related ocular infections are an important limitation of the success of such procedures. Moreover, many infections progress to secondary permanent sequelae that may lead to poor visual outcomes and occasionally loss of sight, such as acute bacterial endophthalmitis or corneal ulceration.

**Table 1:** Biofilm-associated infections of the eye.

Disease	Main Causative Agents of Infection and/or Found in the Biofilms	Biofilm Localization
Endophthalmitis	Coagulase negative staphylococci and *Propionibacterium acnes*	Intraocular lens
		Posterior capsule
Keratitis	*Staphylococcus aureus* and other staphylococcal species, *Pseudomonas aeruginosa* and *Serratia* spp. Fungi and *Acanthamoeba* less frequently	Contact lens
	Viridans group streptococci. Gram negative bacilli and yeasts less frequently	Corneal stroma (crystalline keratophaty)
Scleral buckle infection	Gram positive cocci and nontuberculous *Mycobacterium* ^1^	Scleral buckles
Lacrimal system infections	*Staphylococcus* spp., *P. aeruginosa and M. chelonae*	Lacrimal intubation devices
	*Staphylococcus* spp ^2^	Punctual plugs
Periorbital infections	*Staphylococcus* spp. and mixed species biofilms	*Sockets* and orbital plates

^1^ Common causative agents of buckle-associated infections. Scleritis resulting of scleral extension of corneal infections are mainly caused by *P. aeruginosa* and other common agents of infectious keratitis; ^2^ The presence of biofilms has not been demonstrated on plugs recovered from symptomatic eyes presenting with dacryocystitis and canaliculitis. However, clinical features of these infections are compatible with biofilm-associated infections such as the late onset, and difficulty to treat with antimicrobial therapy alone.

## 4. Biofilms in Endophthalmitis

Endophthalmitis is a rare but severe intraocular inflammation that results from the introduction of a microbial pathogen into the posterior segment of the eye. Organisms may gain access to the intraocular tissues exogenously after trauma caused to the ocular globe following intraocular surgery, intravitreal injections, penetrating open globe injury, and in cases of keratitis progressing to corneal perforation. Endogenous endophthalmitis may occur in patients with bacteremia by seeding the eye with bacteria from a distal site of infection [40].

### Endophthalmitis Following Cataract Surgery

Postoperative endophthalmitis is the most common presentation and is frequently associated with cataract surgery [41,42]. It is estimated that 17.2% of the population older than 40 years in the United States suffers from cataracts. This prevalence increases with age, being more than 35% for patients between 70–74 and almost 50% for patients between 75–79 years of age [43]. As a result, cataract extraction with replacement of the crystalline lens by an artificial intraocular lens (IOL) represents the most frequent surgery procedure performed by ophthalmologists, with more than 1 million procedures performed each year in United Stated [44]. Postoperative endophthalmitis is the leading blinding complication of cataract surgery. Its overall incidence varies according to the technique and region of the world, ranging from 0.028% to 0.2% [45,46,47]. Despite the low overall incidence, given the large number of cataract surgeries performed annually, a substantial number of patients are affected by this sight-threatening infection.

Post-cataract endophthalmitis is caused predominantly by Gram positive organisms originating from the ocular surface microbiota. Coagulase-negative staphylococci (CoNS), especially *Staphylococcus epidermidis*, are the most frequently encountered microbes from culture-proven acute endophthalmitis [41,42,48]. Delayed-onset endophthalmitis is mainly caused by *Propionibacterium acnes*, which usually presents with a more indolent and persistent infection, with lower frequency of hypopyon and better final visual outcome compared to acute cases [49]. Both pathogens, *S. epidermidis* and *P. acnes,* are able to adhere to, and form biofilms on intraocular lenses. Some evidences suggest that they can also adhere to and form biofilms in the posterior capsular bag [50,51,52,53,54,55,56,57,58]. Commensal organisms colonizing the ocular surface and periocular tissues are the primary source of bacteria that cause postoperative endophthalmitis. In the large Endophthalmitis Vitrectomy Study, 67.7% of paired CoNS isolates from the eyelid and intraocular fluids were indistinguishable by pulsed field gel electrophoresis [59].

The ocular surface is often colonized by Gram positive organisms, with CoNS being most commonly associated with healthy conjunctiva, lids and tears, followed by *Propionibacterium acnes*, *Corynebacterium* spp. and *S. aureus* [60]. Rates of contamination of the anterior chamber after uneventful cataract surgery range from 2% to 46%, and are due to the most common Gram positive commensal organisms found on the ocular surface, most frequently *S. epidermidis* [61,62,63,64]. Rates of anterior chamber contamination are much higher than the incidence of postoperative intraocular infection. This suggests that in most cases, the anterior chamber is capable of clearing the bacterial inoculum without progressing to endophthalmitis, likely due in part to the rapid turnover of the aqueous humor [65]. However, most of the organisms found in the ocular microbiota are able to attach to the IOL and posterior lens capsule, and often become well established if they reach the posterior chamber. In comparison to aqueous humor, vitreous is relatively static and constitutes a good environment for establishment of an infection. Intraocular lenses, such as those constructed from polymethylmethacrylate (PMMA) may become contaminated with commensal conjunctival bacteria (mainly *S. epidermidis*) during insertion, and carry the organisms from the ocular surface to the posterior chamber [66,67]. The ability of commensal bacteria to form biofilms on the surface of IOLs prevents their clearance, and likely represents an important mechanism in the pathogenesis of post-cataract endophthalmitis. The occurrence of microbes in biofilms is consistent with the low rates of culture positivity for aqueous and vitreous samples [68].

As described above, the *ica* locus and other genes play an integral part in staphylococcal biofilm formation. One report from Japan found a prevalence of 60% and 69.4% for *ica*A positive strains of *S. epidermidis* isolated from the conjunctiva of healthy volunteers and patients undergoing cataract surgery, respectively. Most of these isolates (approximately 40%) tested positive for slime production on Congo red agar [29]. Among a collection of *S. epidermidis* isolates from Mexico, 17% of commensal conjunctival isolates were able to form biofilms under conditions used *in vitro*, and 26.7% were positive for the *ica*A and/or *ica*D genes [27]. The frequency of *ica* genes was observed to be 36% for CoNS species other than *S. epidermidis* recovered from the normal conjunctiva of student and staff eyes at an institute in India [28]. For *S. epidermidis* recovered from intraocular fluids of patients with endophthalmitis, the distribution of *ica*A and *ica*D genes seemed to be group specific. Among strains isolated from endophthalmitis cases in South Florida, *ica*AD genes were present (86%) only among isolates that possess the accessory gene regulator locus (*agr*) type I. The frequency of *aap* and *bhp* genes among all isolates was 78.5% and 43.1%, respectively, with *bhp* being more prevalent among *agr* type II isolates [69].

**Figure 1:**
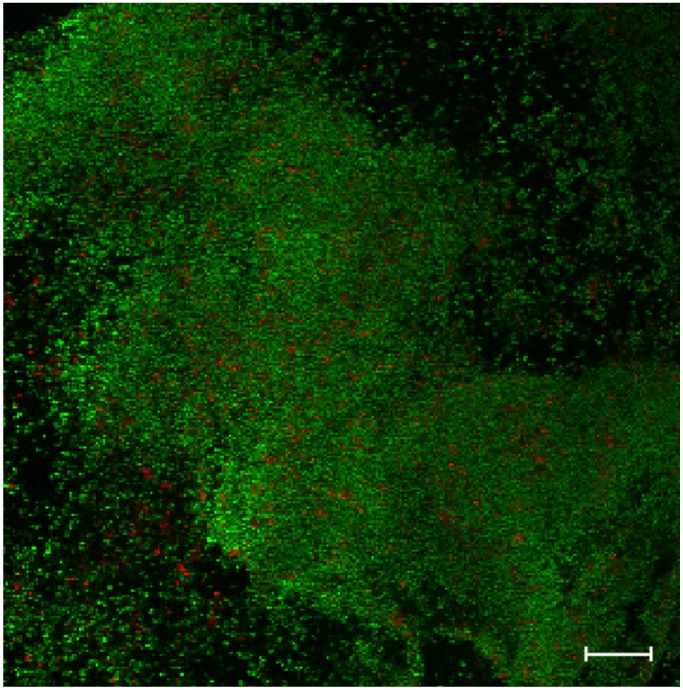
Confocal laser scanning micrograph of a 24 h biofilm. The biofilm of *Staphylococcus epidermidis* RP62A was grown *in vitro* on hydrophilic acrylic intraocular lens, and was visualized after staining using the live/dead viability stain, which contains SYTO9 (green fluorescence, live cells) and propidium iodine (red fluorescence, bacterial cells that have a defective cell membrane, which is indicative of dead cells). Magnification X 400, scale bar is 20 µm.

Previous reports have demonstrated the ability of *S. epidermidis* to form biofilms on IOLs (Figure 1) using different *in vitro* conditions [53,54,55,57], and in a model that resembles the intraocular environment [51,52]. The degree of biofilm formation is affected by the material used to manufacture the IOL and also by the genomic content of each *S. epidermidis* lineage tested. Strains of *S. epidermidis* carrying the *ica* locus are able to form stronger biofilms on different IOL surfaces compared to strains lacking this locus [53,55,57]. In one study [55], using various hydrophobic IOLs, the ability of *S. epidermidis* strains ATCC 12228 (*ica* negative) and ATCC 35984 (*ica* positive) to form biofilms was significantly higher on acrylic lenses followed by PMMA and MPC (2-methacryloyloxyethyl phosphorylcholine) surface-modified acrylic. Weaker biofilms were found on silicone IOLs. Interestingly, modification of the acrylic IOL surface by treatment with MPC decreased biofilm formation [55] and this may be associated with an increase in the hydrophilicity [70]. The same effect has been demonstrated for MPC-modified silicone IOL [71]. Other reports [51,53,72,73], however, have found different results for each IOL material and that was also affected by the strains tested. Foldable IOLs made with silicone supported greater *S. epidermidis* biofilm formation compared to PMMA IOL for strain ATCC 35984, but the same was not seen for the strain ATCC 12228 [53]. In the same study, variations of up to two orders of magnitude in the degree of biofilm formation, as determined by CFU counting, were observed for the same IOL material depending on different models and manufacturers. Acrylic lenses were again the most prone to form stronger biofilms and fluorine-treated PMMA the least. The presence of polypropylene haptics in the PMMA IOL increased the biofilm quantity compared to single-piece PMMA IOL [53]. In agreement, it has been demonstrated that polypropylene haptics represents a significant risk factor for post-cataract surgery endophthalmitis [72], and increases *in vitro* adhesion of *S. epidermidis* compared to single- and three-piece PMMA IOL [73]. In a model using a bioreactor with flow conditions similar to the anterior chamber, *S. epidermidis* was able to form biofilms on different IOL materials, which significantly increased as a function of time [52]. Silicone was more permissive to biofilm formation in this model, followed by hydrophobic acrylic and PMMA, with the fewest attached cells found on hydrophilic acrylic.

Despite the variations within each study, hydrophobicity is consistently observed to be an important determinant of biofilm formation. Modification of the surface to make it more hydrophilic may reduce initial binding and development of robust staphylococcal biofilms. The initial adherence of *S. epidermidis* to the IOL surface, an important initial step for subsequent colony expansion and biofilm maturation, has been demonstrated to be decreased by IOLs with hydrophilic surfaces [74,75]. Surface modifications that increase an IOL’s water content have been made using different agents including MPC, fluorine and heparin. While MPC and fluorine have been demonstrated to decrease the density of biofilms formed on PMMA and silicone IOLs [53,55,71], data for heparin-modified lenses are mixed. Initial adhesion of *S. epidermidis*, *S. aureus* and *P. aeruginosa* to heparin-surface-modified (HSM) PMMA IOL has been shown to be reduced compared to non-treated PMMA IOLs [76,77]. However, biofilm formation on HSM PMMA IOLs seems not to be affected, and in fact biomass measures were higher compared to non-treated PMMA [53]. In addition, although the anti-adhesive effect of soluble heparin in the media has been demonstrated *in vitro* using *S. epidermidis* and PMMA IOLs [78], this protective effect was not demonstrated *in vivo* by the addition of low molecular weight heparin to the infusion fluid used during the phacoemulsification procedure [79].

Although infrequent, enterococcal endophthalmitis may occur after cataract surgery and is associated with poor visual outcomes even after appropriate clinical and surgical management [80]. *In vitro* studies have demonstrated that *Enterococcus faecalis* is able to form robust biofilms on IOLs, especially on PMMA and hydrophobic acrylic IOLs after 48 h and 72 h of incubation, while less biomass was observed on silicone IOLs [81]. Recurrent cases of post-cataract endophthalmitis caused by *E. faecalis* have been associated with bacteria attached to the capsular bag and acrylic IOL [82,83].

While acrylic IOLs have demonstrated to be the most permissive material for biofilm formation of Gram positive pathogens, as described above, this material seems to be less susceptible to adherence and biofilm formation of *Pseudomonas aeruginosa*, compared to PMMA and silicone [84]. This is consistent with the finding that the surface of *P. aeruginosa* is highly hydrophobic, while that of staphylococci is highly hydrophilic [17]. Although *P. aeruginosa* does not represent an important organism associated with endophthalmitis after uneventful cataract surgery, it has been associated with multiple outbreaks of post-cataract surgery endophthalmitis, usually due to environmental contamination including the internal tubes of phacoemulsification machines and contaminated solutions used during the surgery [85]. In a recent outbreak of *P. aeruginosa* endophthalmitis following cataract surgery, a thorough investigation identified the hydrophilic acrylic IOL implanted in the patients and the preservative solution as the source of the contamination. The *P. aeruginosa* isolates from the IOL and the preservative solution had the same genetic profile as the isolates from the patients’ aqueous and vitreous fluids, as demonstrated by ERIC-PCR [86]. In the context of *P. aeruginosa* endophthalmitis outbreaks after cataract surgery, biofilm formation has not been directly associated in the pathogenesis of these infections, but it likely plays a role as a reservoir for contamination. Biofilms found in the hospital environment have been demonstrated to be a common source of *P. aeruginosa* associated with outbreaks in intensive care units [87,88]. *P. aeruginosa* evolved to form biofilms on surfaces in contact with water, including sinks, water pipes and other natural interfaces [89], and this property is undoubtedly central to its ability to colonize the surgical equipment and irrigation fluids that have been linked to endophthalmitis outbreaks.

## 5. Biofilms in Keratitis

Microbial keratitis is an infection of the cornea that can lead to loss of vision if not carefully managed [90]. Decades ago, most cases were associated with ocular surface disease and trauma. However, the widespread of contact lenses has made them the most common predisposing risk factor for infectious keratitis [91,92]. The type of organisms causing keratitis varies by geography because of differences in climate, environment and occupational risk [93]. Bacterial keratitis, especially contact lens-associated infection, is caused by both Gram negative pathogens, such as *P. aeruginosa* and *Serratia* spp., and Gram positive organisms, such as *S. aureus* [94,95] and other staphylococcal species [93,96,97]. Risk factors for fungal keratitis include tropical or subtropical climate, agricultural work, and corneal trauma [93,98]. In the United States, the prevalence of fungal keratitis is much lower than bacterial keratitis [99], and the primary predisposing factor is unambiguously contact lens wear [100,101]. When it does occur, *Fusarium* spp. usually accounts for the majority of fungal keratitis cases [101]. Additionally, *Acanthamoeba* are protozoa that cause a rare but aggressive form of infectious keratitis that is also frequently associated with contact lens wear [102].

### 5.1. Contact Lens-Associated Keratitis

Contact lens use represents the main risk factor for the development of microbial keratitis in developed countries, where it is associated with bacterial, fungal and amoebic keratitis [91,92,100,101,102,103]. In the United States, previous estimates of microbial keratitis suggested more than 30,000 cases per year [104]. Other estimates of ulcerative keratitis in northern California found an incidence of 27.6 cases per 100,000 person/year [103]. This incidence was higher than observed previously, and was thought to be associated with increasing contact lens wear, since the rate of keratitis was half of that for non-contact lens wearers [103].

The incidence of contact lens-associated microbial keratitis has been shown to be impacted by the contact lens material, and also by the wear schedule. Early epidemiological studies reported a higher risk for daily wear soft contact lenses compared to daily wear rigid gas permeable lenses. The risk was further increased for extended wear (overnight wear) soft contact lenses [95,105]. More recently introduced daily disposable and silicone hydrogel contact lenses have also been associated with a higher incidence of keratitis compared to rigid gas permeable contact lenses [106,107].

The increased risk for the development of microbial keratitis in contact lens wearers has been associated with the ability of the lens to induce modification of the corneal epithelium, to carry organisms to the ocular surface that otherwise would not be found in this niche, and to limit natural clearance mechanisms [108,109]. The close interaction between the lens and the corneal epithelium induces local alterations, including hypoxia and hypercapnia, which affect the ability of the epithelium to respond to damage. Tear fluid exchange may be compromised between the anterior and posterior sides of the lens, altering the composition of the tear fluid on the ocular surface and limiting its antimicrobial properties [108]. In addition, contact lenses provide a surface where microorganisms may attach and colonize the surface as a biofilm, which represents a source for microorganisms to spread to a previously damaged corneal epithelium [109]. It has been demonstrated that poor hygiene and infrequent replacement of the contact lens storage cases were independent risk factors for moderate and sever keratitis [110]. However, not all individuals with poor contact lens hygiene will experience keratitis, while others with good cleaning routines also suffered infections, suggesting that other factors play a role as well [111].

Corneal damage promotes colonization and infection by commensal and environmental organisms. The high prevalence of Gram negative organisms among contact lens-associated keratitis isolates, which are usually not found as commensals on the ocular surface, is likely due to their ubiquitous presence in the environment and their ability to adhere and subsequently form biofilms on the surface of contact lenses and storage cases. *P. aeruginosa* has a repertoire of genes that allow its adaptation and survival under different stress conditions [112]. Its ability to adhere to different contact lens materials has been demonstrated *in vitro* [113] and is mainly driven by surface hydrophobicity [17,114]. Development of mature biofilms in the posterior surface of the contact lens has been associated with *P. aeruginosa* keratitis in humans [115] and in animal models [31].

In addition to bacteria, fungi and *Acanthamoeba spp*. are also able of causing contact lens associated keratitis. Some of these cases were linked to specific multipurpose contact lens cleaning solutions (MPS), which led to the removal of these products from the market. The first case involved the fungus *Fusarium solani*, which was associated with keratitis in patients that used ReNu with MoistureLoc. This finding came as a surprise because all MPS have to pass antimicrobial efficacy testing against several microorganisms, including *F. solani*. An investigation [116] found that planktonic *Fusarium* strains were susceptible to MoistureLoc as expected, but *Fusarium* biofilms showed reduced susceptibility. In addition, *F. solani* ATCC 36031, the reference strain recommended for antimicrobial efficacy testing, was shown to be incapable of forming biofilms under the conditions tested [116]. This case highlights the need to consider the physiological state of microbes and the strains used in establishing testing standards—standards largely developed using planktonic cells. The second case involves Complete MoisturePlus and protozoa of the genus *Acanthamoeba*. These amoebae are ubiquitous in water and are able to survive harsh conditions, including chemical treatment, by differentiating into dormant cysts that can resume growth once favorable conditions return. Bacterial biofilm formation on contact lenses is a risk factor for contact lens-associated keratitis by *Acanthamoeba* because these organisms graze on the biofilm [117,118,119]. Complete MoisturePlus was recalled because, rather than killing all cells, it resulted in the encystment of *Acanthamoeba*, which then went on to cause keratitis [120]. Prior to this recall, antimicrobial efficacy testing that included *Acanthamoeba* was not required [121].

### 5.2. Infectious Crystalline Keratopathy

Crystalline keratopathy is a disease associated with crystalline deposits in the corneal epithelium and stroma, which may be a result of multiple conditions that ultimately lead to the accumulation of metabolic products in the affected corneal tissue. Among the causes of such deposits are corneal infections and systemic diseases [122]. Infectious crystalline keratopathy (ICK) is a chronic and difficult to treat infection of the cornea characterized by the presence of branching crystalline opacities associated with minimum inflammatory response [123,124]. It may occur in normal or diseased corneas and is often associated with corneal surgery, especially penetrating keratoplasty, and the topical use of steroids [122,124,125]. Viridans streptococci are the main pathogens associated with ICK, but other bacterial and fungal species, as well as *Acanthamoeba*, may cause this infection [125,126,127,128].

Because of the indolent clinical evolution of this disease, and the relative lack of immune response and recalcitrance to antimicrobial therapy, it was first hypothesized that ICK resulted from organisms associated with a biofilm in the corneal tissue. This was supported by analysis of corneal samples collected by biopsy or penetrating keratoplasty from patients with ICK [129,130,131]. Transmission electron microscopy examination of corneal samples fixed with ruthenium red found *Candida albicans* and also bacteria surrounded by an extracellular matrix consistent with a biofilm [129,130]. Microscopic evidence of bacterial biofilms growing on the corneal tissue was also found for 3 patients diagnosed with ICK, but for 5 other cases of chronic bacterial and fungal keratitis. This indicates that *in vivo* biofilm formation in the corneal stroma is fairly specific for ICK [130]. The intensity of the periodic acid-Schiff stain of the corneal samples from ICK [130] is indicative of high concentrations of polysaccharides, often a main factor associated with the ability of bacteria to form a strong and well organized multicellular structure. The presence of extracellular polysaccharides in abundant amounts also has been observed in a histologic analysis of corneal sections from rabbits with crystalline lesions induced by a *Streptococcus sanguis* type II strain, but were absent in the eyes that developed suppurative stromal lesion [132]. Interestingly, this study showed that *S. sanguis* grown in medium supplemented with sucrose produced exopolysaccharides, resulting in a mucoid phenotype and a crystalline lesion similar to ICK in 71% of the corneas inoculated. In contrast, strains grown without sucrose showed a rough phenotype, caused crystalline lesions in only 25% of the eyes, and were more frequently associated with suppurative infiltrates [132].

It is unclear what cues induce microbes to grow as planktonic cells or invade tissues and form a biofilm in ICK. Prolonged topical corticosteroid therapy and/or prior penetrating keratoplasty have been identified as risk factors for the development of ICK in the vast majority of patients [123,124,125,126,128,129,130,131]. Anatomical modification from the keratoplasty procedure, often resulting in inflammation and altered local immune activity, appears to predispose microbes to grow in a biofilm, but the underlying mechanism has not been thoroughly explored.

As for other biofilm-related infections, antimicrobial treatment of ICK is challenging in that it is usually prolonged and the disease is often unresponsive. Physical means have been explored for improving the success of ICK treatment, including the use of laser disruption of biofilms [133,134]. In this case, Nd:YAG (neodymium-doped yttrium aluminum garnet) laser photocoagulation was used to disrupt the crystalline deposits in the cornea of patients unsuccessfully treated with antibiotics. In all cases, laser disruption with further antimicrobial therapy resolved ICK within weeks, and no recurrence was observed.

## 6. Biofilms Associated with Other Implant-Related Ocular Infections

### 6.1. Scleral Buckles

The placement of permanent scleral buckles between the conjunctiva and sclera is a common surgical treatment for rhegmatogenous retinal detachment. The bands encircling the sclera are commonly made of silicone and may have a solid or sponge form. One of the main complications associated with this surgery is the extrusion of the bands, which is frequently associated with an infection. Scleral buckle-associated infections are frequently caused by Gram positive cocci, especially coagulase-negative staphylococci, and nontuberculous *Mycobacterium* [135,136]. These infections often have a delayed onset and are usually refractory to antimicrobial therapy, requiring removal of the bands for complete resolution [135]. Due to the chronic evolution of this infection, the presence of a biofilm in the explanted material has been assumed to play an important role in its pathogenesis. Biofilms have been demonstrated by scanning electron microscopy for 65% of scleral buckles (solid and sponge forms) removed for infection and extrusion [137]. Gram positive bacteria, and less frequently *Mycobacterium chelonae* and *Proteus mirabilis,* were cultured from these buckle elements [137]. Buckle materials removed from patients because of conjunctival erosion and infection, or due to technical reasons at the time of revision surgery, contained demonstrable bacterial biofilms in 5 out 28 cases examined by scanning electron microscopy following fixation with ruthenium red [138]. Of those five, one was removed due to extrusion, and one was associated with a diagnosed infection. The three remaining buckles were removed for other reasons from patients lacking signs of infection. It seems likely that at the time of surgery, bacteria attach to the buckle material and form biofilms that lead to indolent infections, or infections lacking any overt signs.

### 6.2. Conjunctival Plug

Punctual plugs are frequently used to treat ocular surface dryness unresponsive to topical medication, by occluding the lacrimal ducts and blocking tear drainage. Plugs are made of silicone, hydrophobic acrylic, collagen and hydrogel. However, secondary complications may occur following implantation, including canaliculitis, dacryocystitis and acute conjunctivitis [139,140]. Although the presence of biofilms has not been demonstrated on plugs recovered from patients with dacryocystitis and canaliculitis, these infections typically have a late onset and are usually not responsive to antimicrobial treatment alone, requiring additional intervention, as is typical for biofilm-related infections [141,142]. Examination of punctual plugs removed from patients without clinical signs of infection revealed the presence of bacterial biofilms in 53% of the samples assessed by electron microscopy [143]. However, since the patients were asymptomatic with respect to infection, the causal association between the biofilms growing on punctual plugs and progress to an eye infection remains speculative. A single case of conjunctivitis has been associated with biofilm formation on a punctual plug [140]. That patient presented with acute conjunctivitis in the same eye that had received a punctual plug five and half months earlier. A whitish material that was culture positive for *S. haemolyticus* and *Candida tropicalis* was present in the top of the plug. Analysis of this plug by scanning electron microscopy demonstrated the presence of a bacterial biofilm. The plug was removed and the patient resolved the infection after 1 month of topical antimicrobial treatment [140].

### 6.3. Lacrimal Intubation Devices and Orbital Implants

Lacrimal intubation devices including lacrimal stents and Jones tubes are commonly used during the dacryocystorhinostomy procedure to treat nasolacrimal duct obstruction, a common cause of epiphora. As for other biomaterials implanted in the eyes, both lacrimal stents and Jones tube may provide with a surface for biofilm formation [144,145,146]. Biofilm formation on polyurethane nasolacrimal stents has been associated with delayed failure of the device [145]. In a study undertaken to identify the rates of biofilm colonization on silicone stents inserted during dacryocystorhinostomy, 90% of stent fragments removed 8 weeks after surgery revealed the presence of coccoid and/or rod-shaped bacteria encased in a biofilm matrix [144]. All patients included in this last study had received postoperative antibiotics for 1 week (oral) and 3 weeks (topical) and the silicone tubes were collected at the 8th week post-surgery. Most of these silicone stents were culture positive for *S. epidermidis* and *P. aeruginosa* [144].

A report of 2 cases of nasolacrimal infection following placement of a lacrimal silicone and a Jones tube described recalcitrant culture-negative infections associated with the presence of bacterial biofilms in the lacrimal intubation devices [147]. Evaluation of both silicone stent and Jones tube by scanning electron microscopy revealed the presence of a polymicrobial biofilm. Interestingly, the authors were able to identify a variety of cell morphologies in the silicone stent biofilms, including short rods, spirochetes, fusiforms and cocci. Analysis of the internal surfaces of the silicone stent by confocal laser scanning microscopy revealed the presence of viable biofilms along the tube [147].

In a series of cases with recalcitrant infections associated with silicone stents (*n* = 10) and Jones tube implants (*n* = 2), a high prevalence of *Mycobacterium chelonae* (90%) was found associated with the silicone stents, sometimes along with other bacterial organisms [146]. In this same study, the authors evaluated the culture results and presence of biofilms in other periorbital biomaterials, including orbital plates (*n* = 5) and anophthalmic socket sphere implants (*n* = 4) [146]. Cultures were positive for *S. aureus* in all orbital spheres, in addition to one isolate of *M. chelonae* and one *Pantoea agglomerans* that were found in polymicrobial cases. Culture results of orbital plates demonstrated more species diversity with the isolation of yeasts (*Candida* spp., and *Trichosporon* spp.), *Staphylococcus* spp., *M. chelonae* and Gram negative bacilli (*Achromobacter xylosoxidans* and *P. aeruginosa*). Scanning electron microscopy analysis of selected samples also demonstrated the presence of mixed species biofilms on porous polyethylene orbital floor implant, metal screws from orbital plate implant and on orbital sphere implants [146].

## 7. Perspectives on Agents for Prevention and Treatment of Biofilms

Since biofilms have been recognized for their great medical importance, efforts have been made to either prevent their formation, or to remove them once they have formed. The colonization of surfaces can be prevented by covalently attaching biocidal molecules, slowly releasing antibiotics, or modifying the surface topology to interfere with microbial adhesion. While the first two approaches would be practically easier to achieve as it depends basically on coating current ocular devices with available molecules, the last may be more challenging as modifications in the topology of the material may alter its optical clarity. However, both approaches have their advantages and disadvantages. As an example for prevention of surface colonization using biocides, in a previous study authors have demonstrated a 100-fold reduction in cell counts when *S. aureus*, *S. epidermidis*, *E. coli*, or *P. aeruginosa* were sprayed onto glass slides that were coated with covalently attached poly(4-vinyl-*N*-alkylpyridinium bromide) or *N*-hexylated poly(4-vinylpyridine) [148]. Surface coatings that slowly release antibiotics, such as rifampin, clarithromycin and doxycycline, were able to prevent biofilm formation for up to three weeks *in vitro* [149]. An IOL designed to release norfloxacin to prevent postoperative bacterial infection after cataract surgery has been tested *in vitro* and in a rabbit model, and might soon become commercially available [150]. Antimicrobial peptides have also been used successfully to prevent biofilm formation and have the added advantage that they are active against antibiotic-resistant strains [151]. Other molecules that can prevent the formation of biofilms, such as gallium nitrate or silver, have also shown potential [151]. While molecules that are slowly released from a surface to prevent biofilm formation show great promise for some applications, questions remain regarding their use in medical devices. The concentration of the antimicrobial agent would have to remain sufficiently high as long as colonization and infection is a risk, and not select for resistant strains. In addition, lack of toxicity and long-term compatibility with surrounding tissues is important [152]. The advantages of using ocular devices that slowly release antimicrobial agents is that tissue toxicity, penetration and half-life is already known for a number of antibiotics routinely used in ophthalmology. On the other hand, long-term exposure to these drugs may favor selection of spontaneous resistant mutants and perturbs the ocular surface microbiome.

Modifying the surface structure of ocular devices to make it less adhesive for bacteria attempting to colonize is a tempting approach since it would potentially eliminate the need for coating with antimicrobial or biocide agents that could be reserved for treatment and perioperative prophylaxis. However, alterations in the material topology that results in material opacities may limit its use for optical correction. Polymers, such polyacrylamide, dextran, or polyethylene glycol, can form linear, star-shaped, or ‘bottle brush’ shaped surface nanostructures that interfere with the microbe’s ability to adhere to the substrate [153,154]. Nanopores, nanotubes, and nanopillars made of anodized aluminum, titanium dioxide, or polymethylmethacrylate have also been investigated to reduce microbial adhesion to coated surfaces [152]. Even low-fouling substrates may eventually become colonized due to degradation or erosion of the anti-adhesive surface [154], so this will have to be explored. Furthermore, some medical devices require the firm binding of the implant to the surrounding tissue for optimal biointegration, which may limit the use of this strategy in some cases. Other devices, such as contact lenses, may be difficult to modify this way without negatively affecting critical properties, such as optical clarity.

An alternative strategy for the removal of microbial biofilms is to stimulate the reversion of microbes to planktonic physiology. While the enzymes that degrade the ECM or the substratum might be too large and costly to be of practical clinical value, small signaling molecules that induce expression of factors that stimulate the dissimilation of biofilms might be a viable alternative. Cell signaling molecules, such as C4HSL, PQS, AI-2, and AIP-I or their derivatives, may be of great therapeutic value [35,38].

While typical antibiotics work well against growing planktonic cells and are less active, or inactive, against the dormant cells in a biofilm, they may still have a use in combination therapies. The acyldepsipeptide antibiotic, ADEP4, was shown to bind to the ClpP protease of *S. aureus* and convert it into a nonspecific protease that degrades over 400 proteins, killing growing as well as dormant cells [155]. Treatment with ADEP4 resulted in the emergence of ADEP4-resistant *clpP* mutants, but those were highly susceptible to killing by various antibiotics. Using a deep-seated mouse biofilm infection model, the authors showed that ADEP4 in combination with rifampicin was able to reduce the number of *S. aureus* below detectable limits, while neither rifampicin, vancomycin, nor ADEP4 by themselves were able to do so [155]. The use of bacteriophage endolysins as well as engineered phages expressing anti-biofilm enzymes may also be promising options for eradication of bacterial biofilms in the site of infection [156,157]. Evolutionary distinct bacteriophage endolysins have shown to be effective in killing planktonic cells as well biofilms of *S. aureus* and prevented death of 100% of mice inoculated intraperitoneally with lethal doses of MRSA [157]. A T7 bacteriophage engineered to express dispersin B (DspB), an enzyme that hydrolyzes β-1,6-N-acetyl-d-glucosamine, has been successfully used to simultaneously infect and kill the bacterial cells in the biofilm, in this case *E. coli*, and also attack the extracellular polymeric biofilm matrix [156].

The field of biofilm dispersal and eradication is an area of very active research and the next five years will see a substantial increase in our understanding of the physiological states associated with biofilms. It is hoped that this will lead to the development of new agents that will pass clinical trials and serve as new treatments for microbial infections caused by cells in a biofilm state.

## 8. Conclusions

Our understanding of biofilms has advanced substantially since early descriptions more than three decades ago. As medical interventions rely increasingly on medical devices and prosthesis, the need to prevent, reduce, or eliminate microbial biofilms is becoming an important constraint. In the eye care field, contact lenses and IOLs have had a great impact on restoring and improving vision, but their use is limited by ocular infection. Strategies, such as anti-biofilm surface coatings and developing biofilm-active therapeutics, are exciting avenues of future research to reduce the risk of biofilm-associated ocular infection.
